# Radiographic evaluation of malpositioning in immediate and early dental implant placement: A cross-sectional study

**DOI:** 10.34172/japid.026.4041

**Published:** 2026-05-06

**Authors:** Fatima Waleed Khalid, Zeinab Kadkhoda, Amir Raee, Farzaneh Mosavat, Shima Younespour

**Affiliations:** ^1^Department of Periodontics, School of Dentistry, Tehran University of Medical Sciences, Tehran, Iran; ^2^Dental Implant Research Center, Dentistry Research Institute, Tehran University of Medical Sciences, Tehran, Iran; ^3^Department of Oral and Maxillofacial Radiology, Faculty of Dentistry, Tehran University of Medical Sciences, Tehran, Iran; ^4^Craniomaxillofacial Research Center, Shariati Hospital, Tehran University of Medical Sciences, Tehran, Iran; ^5^Research Center for Caries Prevention, Dentistry Research Institute, Tehran University of Medical Sciences, Tehran, Iran

**Keywords:** Anatomic landmarks, Dental implants, Implant malposition, Radiography, Treatment timing

## Abstract

**Introduction:**

Dental implants are commonly used to replace missing teeth. Immediate implant placement (IIP) has now become an increasingly common strategy to reduce treatment time. Dental implant malposition occurs when an implant is placed at an inappropriate dimension through the bone, which leads to a variety of aesthetic and functional complications. Therefore, this study aimed to compare immediate implant placement (IIP) and early implant placement (EIP) with respect to malposition.

**Methods:**

This cross-sectional study analyzed existing clinical records and accompanying periapical (PA) radiographs of 67 immediate implant placements (IIP) and 152 early implant placements (EIP). All the implants had originally been placed by senior periodontics residents. Postoperative PA radiographs obtained at the time of surgery were reviewed to assess implant malpositioning. Dental implant malposition was defined as mesiodistal angulation≥15° or inadequate proximity (<1.5 mm to adjacent teeth or<3 mm to adjacent implants). The associations between variables and malpositioning were assessed using both univariate and multivariable logistic regression.

**Results:**

A total of 219 implants were included from 85 individuals, comprising 31 males (36.47%) and 54 females (63.53%), with a mean age of 49.88±14.06 (range: 20‒80). Multivariable logistic regression analysis indicated that both the timing of implant placement and the implant position (maxilla vs. mandible) significantly influenced the risk of malpositioning. IIP was associated with a lower probability of malpositioning than EIP (adjusted OR=0.38; 95% CI: 0.16–0.90; *P*=0.03; Nagelkerke R^2^=0.10).

**Conclusion:**

Within the limitations of this cross-sectional study, multivariable regression analysis indicated that both the timing and position of implant placement are predictors of malpositioning. IIP demonstrated a lower risk of malpositioning compared with EIP, while maxillary placement remained a significant risk factor.

## Graphical Abstract

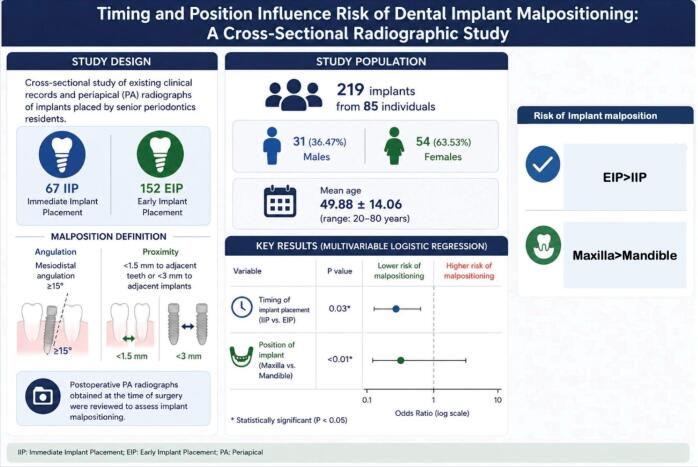

## Introduction

 Dental implant malposition is a clinical complication that can adversely affect treatment outcomes. Tooth loss can lead to functional and aesthetic impairments, underscoring the need for effective treatment planning strategies that support prosthetic restorations supported by dental implants. The success of these restorations depends on the precise positioning of the implants, which is crucial for achieving long-lasting, reliable prosthetic outcomes. The prevalence of dental implants among US adults rose dramatically from 0.7% (1999‒2000) to 5.7% (2015‒2016). Current models project that this figure will reach approximately 17% by 2026.^1^ This rising treatment prevalence will inevitably lead to a corresponding increase in the absolute number of patients experiencing complications, presenting a growing challenge for clinical management.

 The timing of implant placement following tooth extraction represents a key consideration in contemporary treatment planning. To accommodate varying clinical scenarios and patient needs, four implant placement timing protocols have been established as immediate (Type 1: simultaneous with tooth extraction), early (Type 2: 4‒8 weeks after extraction with soft tissue healing), early (Type 3: 12‒16 weeks after extraction with partial bone healing), and late (Type 4: more than 6 months after extraction).^2,3^

 According to history, the first dental implant placement protocols required a 6-month healing period after tooth extraction to allow osseous healing of the post-extraction socket, followed by an additional 3–6 months before dental implant loading.^4^ To mitigate alveolar ridge resorption, the 2013 ITI Consensus Conference protocol recommends avoiding late implant placement and instead advocates for earlier implant insertion following tooth extraction.^5^ Moreover, the patients might be more satisfied with an accelerated treatment strategy.^4^

 The malpositioning of implants is a common issue,^6^ triggering a cascade of aesthetic and prosthetic problems. It can also lead to the invasion of anatomic structures. Patients may experience mucosal marginal recession. Furthermore, implant malposition complicates the prosthodontic phase, challenging the achievement of an optimal emergence profile, passive fit, and long-term aesthetic integration.^7^ Accurate implant positioning consists of accurate mesiodistal, buccolingual, and apicocoronal dimensions. Previous studies have shown that the ideal implant angulation is perpendicular to the crest of the bone in both the buccolingual and mesiodistal axial dimensions. The more an implant is tilted, the greater the stress on the bone crest.^8,9^ Within a 0°‒15° range, stress on implants is more evenly distributed, while bone stress increases dramatically when implant angulation is increased more than 15°.^10,11^ On the other hand, the prosthetic and esthetic complications may also increase in angulated implant positioning. Another critical factor in assessing implant positioning is the mesiodistal spacing. To ensure biological health and aesthetic outcomes, a minimum distance of 1.5 mm should be maintained between an implant and an adjacent natural tooth, and at least 3.0 mm between two adjacent implants.^7^

 Previous studies have focused on dental implant malpositioning and implant positioning complications.^12,13^ To our knowledge, the literature is scarce regarding comparisons between immediate (Type 1) and early (Type 3) implantation approaches regarding malpositioning rates. Schnutenhaus et al.^14^ reported that the timing of implantation significantly influenced the precision of free-hand implant placement, with early placement showing greater angular and positional deviations than late placement. Similarly, Choi et al.^15^ found that immediate implant placement, compared with delayed implant placement, resulted in greater accuracy, particularly in mesiodistal positioning. Therefore, the purpose of this cross-sectional study was to compare the immediate (Type 1) and early implantation (Type 3) approaches regarding the occurrence of malpositioning.

## Methods

 This cross-sectional study was reviewed and approved by the Ethics Committee of Tehran University of Medical Sciences (ID: IR.TUMS.DENTISTRY.REC.1402.071) and was in accordance with the Helsinki Declaration of 1975, as revised in 2008. A total of 219 patients (67 IIP [Type 1] and 152 EIP [Type 3]) were enrolled in the study. A signed, detailed, and informed consent form was obtained from all the patients undergoing the treatment. The inclusion and exclusion criteria were as follows.

###  Inclusion Criteria

 Patients undergoing either immediate or early implantation at the School of Dentistry, Tehran University of Medical Sciences.

###  Exclusion Criteria

1. Parallel periapical radiographs of insufficient diagnostic quality
2. Implants positioned in a ridge with a pronounced mesiodistal slope or a vertical bone defect
3. Patients with tremor diseases, Parkinson’s, etc., possibly interfering with the stable head position during the drilling
4. To avoid compromising the PDL space, cases with adjacent teeth tilted > 15° were excluded. This tilt necessitates changing the drilling pathway from a perpendicular position to the bone crest to safeguard the PDL and tooth structure.

**Figure 1:**
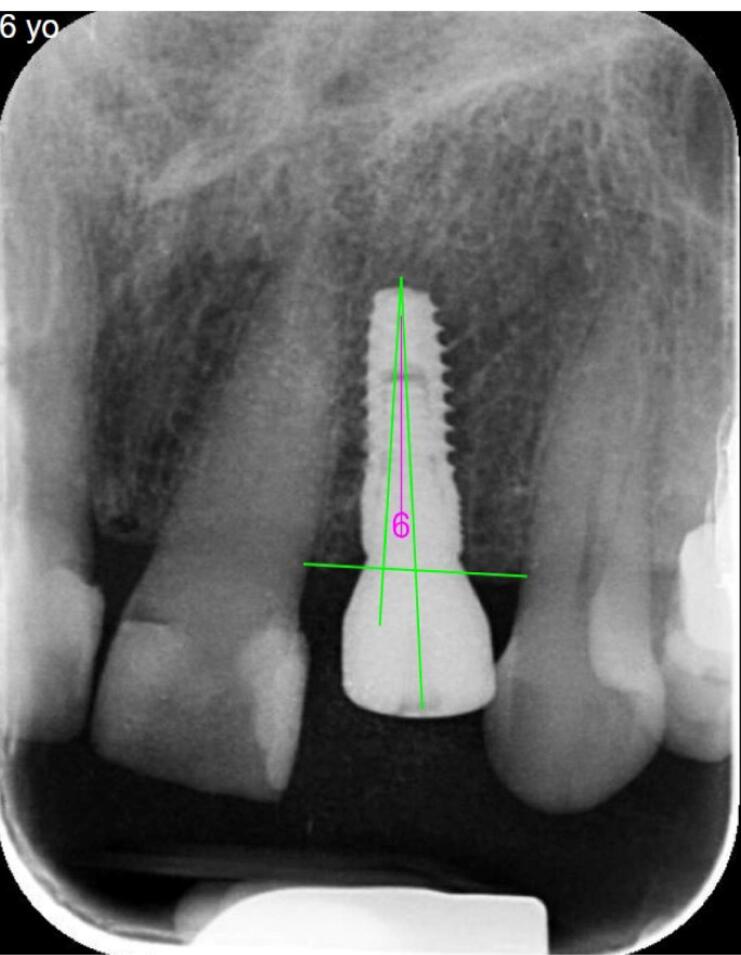
Measurement of the angle formed by the implant’s long axis in relation to the bone crest. The vertical line parallel to the long axis of the implant at the midpoint of the implant was drawn. Another line was drawn perpendicular to the bone crest, and the angle between these lines was measured and evaluated

All eligible patients who met the criteria and were treated at the implant department of Tehran University of Medical Sciences were consecutively enrolled in the study. The surgeries were performed by different senior periodontics residents. Parallel PA radiographs were captured immediately after surgery. All the radiographs were captured using the dental X-ray unit (Owandy RX, France), set to exposure conditions of 60 kVp and 32% mAS. A PSP sensor and parallel XCP were used for the procedure. Radiographs were scanned using the Soredex Digora Optime reader (DIGORA^TM^). To control magnification, all linear measurements were calibrated using the known implant length as a reference. Radiographic assessments were performed twice by the same examiner with a 2-week interval, and intra-observer reliability was confirmed with an intraclass correlation coefficient (ICC) of > 0.90. Medal PACS software was used to assess implant positioning. The Medal PACS software measured the angle formed by the implant’s long axis in relation to the bone crest. A vertical line parallel to the implant’s long axis, passing through its midpoint, was drawn. Another line was drawn perpendicular to the bone crest, and the angle between these lines was measured and evaluated (Figure 1). Additionally, distances between the implant and adjacent tooth (if < 1.5 mm) or between two adjacent implants (if < 3 mm) were measured at the crestal bone. Implants were classified according to the following: anatomical location: anterior maxilla, posterior maxilla, anterior mandible, or posterior mandible; diameter: extra-narrow ( < 3.0 mm), narrow ( ≥ 3.0 mm to < 3.75 mm), standard ( ≥ 3.75 to < 5 mm), and wide ( ≥ 5 mm); length: extra-short ( ≤ 6 mm), short ( > 6 mm to < 10 mm), standard ( ≥ 10 mm to < 13 mm), and long ( ≥ 13 mm); graft at the implant site (i.e., bone graft or bone substitutes): present or absent. Other factors, such as the implant insertion site, were gathered from patient records.

 Implants angled angulation ≥ 15° to the bone crest in mesiodistal axial dimension, or implants located in proximity to an adjacent tooth ( < 1.5 mm) or another implant ( < 3 mm), were considered as the case of malposition. Malposition rate was calculated by dividing the number of mispositioned implants by the total sample size.

###  Data Analysis 

 Statistical analysis was carried out using IBM SPSS Statistics for Windows, version 25 (IBM Corp., Armonk, NY, USA). All the analyses were conducted as two-sided tests, and significance was established at *P* < 0.05. Categorical variables were presented as frequencies and percentages. The normality assumption for the continuous variable was assessed through the Shapiro–Wilk test. Quantitative variables were denoted as mean ± SD or median (25th to 75th percentiles). Univariable and multivariable logistic regression analyses were executed to pinpoint factors linked to the occurrence of malpositioning. The findings were summarized by presenting both crude and adjusted odds ratios (ORs) along with their respective 95% confidence intervals. A multivariabe logistic regression model was constructed using a forward stepwise selection method. Variables with *P* < 0.20 in univariable analyses were entered sequentially into the model, and those with *P* < 0.05 were retained.

###  Sample Size Calculation 

 The sample size was planned based on the comparison of two independent proportions (malposition occurrence in immediate vs. delayed implants) using Pearson’s chi-squared test. In PASS (Version 2023; NCSS, Kaysville, UT), this corresponds to the Z-test (pooled) with the normal approximation. Planning proportions were based on preliminary data from our setting (immediate P₁ = 0.06, delayed P₂ = 0.20; difference: Δ = 0.14), and a two-sided α = 0.05. As recruitment was consecutive, the unequal allocation was fixed to the realized sample sizes (N₁ = 67 immediate, N₂ = 152 delayed). Under these inputs, PASS yielded an achieved power of 0.842 to detect the assumed difference. Thus, the available sample provided adequate statistical power for the primary comparison.

## Results

###  Implant Characteristics

 A total of 219 implants were included from 85 individuals, comprising 31 males (36.47%) and 54 females (63.53%), with a mean age of 49.88 ± 14.06 (range: 20‒80). Table 1 summarizes implant characteristics at various sites of the jaw.

**Table 1:** Implant characteristics at various sites of the jaw

	**Anatomical location**				
**Implant characteristics**	**Anterior maxilla ** **(n=36)**	**Posterior maxilla ** **(n=77)**	**Anterior mandible ** **(n=17)**	**Posterior mandible** **(n=89)**	**Total ** **(n=219)**
Tooth number					
1	8 (22.22%)	-	4 (23.53%)	-	12 (5.48%)
2	17 (47.22%)	-	6 (35.29%)	-	23 (10.50%)
3	11 (30.56%)	-	7 (41.18%)	-	18 (8.22%)
4	-	18 (23.38%)	-	15 (16.85%)	33 (15.07%)
5	-	29 (37.66%)	-	19 (21.35%)	48 (21.92%)
6	-	22 (28.57%)	-	41 (46.07%)	63 (28.77%)
7	-	8 (10.39%)	-	14 (15.73%)	22 (10.05%)
Implant diameter*					
Narrow	23 (63.89%)	27 (35.06%)	10 (58.82%)	20 (22.47%)	80 (36.53%)
Standard	13 (36.11%)	50 (64.94%)	7 (41.18%)	67 (75.28%)	137 (62.56%)
Wide	-	-	-	2 (2.25%)	2 (0.91%)
Implant length^#^					
Extra-short	1 (2.78%)	-	-	3 (3.37%)	4 (1.83%)
Short	3 (8.33%)	17 (22.08%)	1 (5.88%)	35 (39.33%)	56 (25.57%)
Standard	32 (88.89%)	60 (77.92%)	16 (94.12%)	51 (57.30%)	159 (72.60%)
Long	-	-	-	-	-
Timing of implant placement					
Immediate implant placement	19 (52.78%)	21 (27.27%)	11 (64.71%)	16 (17.98%)	67 (30.59%)
Early implant placement	17 (47.22%)	56 (72.73%)	6 (35.29%)	73 (82.02%)	152 (69.41%)
Additional treatment					
Guided bone regeneration	5 (13.89%)	15 (19.48%)	5 (29.41%)	15 (16.85%)	40 (18.26%)
Bone expansion	2 (5.56%)	-	1 (5.88%)	1 (1.12%)	4 (1.83%)
Close sinus floor elevation	1 (2.78%)	2 (2.60%)	-	-	3 (1.37%)
Open sinus floor elevation	-	3 (3.90%)	-	-	3 (1.37%)
Ridge reduction	-	-	3 (17.65%)	-	3 (1.37%)
Connective tissue graft	-	-	-	1 (1.12%)	1 (0.46%)
No additional treatment	28 (77.78%)	57 (74.03%)	8 (47.06%)	72 (80.90%)	165 (75.34%)
Proximity to adjacent tooth					
Yes	2 (5.56%)	3 (3.90%)	1 (5.88%)	3 (3.37%)	9 (4.11%)
No	34 (94.44%)	74 (96.10%)	16 (94.12%)	86 (96.63%)	210 (95.89%)
Proximity to adjacent implant					
Yes	-	-	-	-	-
No	36 (100.00%)	77 (100.00%)	17 (100.00%)	89 (100.00%)	219 (100.00%)
Implant angulation, degree					
Median (IQR)	6 (2.25 to 13.5)	8 (4 to 17)	5 (4 to 7.5)	5 (2 to 9.5)	6 (3 to 11)
Range	(0 to 33)	(0 to 36)	(3 to 13)	(0 to 22)	(0 to 36)
< 15°	32 (88.89%)	54 (70.13%)	17 (100.00%)	81 (91.01%)	184 (84.02%)
≥ 15° to < 20°	2 (5.56%)	10 (12.99%)	-	7 (7.87%)	19 (8.68%)
≥ 20°	2 (5.56%)	13 (16.88%)	-	1 (1.12%)	16 (7.31%)

Note: Data are expressed as no. (%), unless otherwise stated. Abbreviations: IQR, interquartile range (25th to 75th percentiles). *Implant diameter: extra-narrow ( < 3.0 mm), narrow ( ≥ 3.0 to < 3.75 mm), standard ( ≥ 3.75 to < 5 mm), and wide ( ≥ 5 mm) #Implant length: extra-short ( ≤ 6 mm), short ( > 6 to < 10 mm), standard ( ≥ 10 to < 13 mm), and long ( ≥ 13 mm)

 Overall, proximity to adjacent teeth, proximity to adjacent implants, and malpositioning were 9 (4.11%), 0 (0%), and 43 (19.63%), respectively. The distribution of implant malpositioning based on implant characteristics was as follows: 7 (13.21%) in the anterior site, 36 (21.69%) in the posterior site; 31 (27.43%) in the maxilla, 12 (11.32%) in the mandible; 15 (18.75%) in narrow implants, 28 (20.44%) in standard + wide implants; 8 (14.28%) in extra-short + short implants, 35 (22.01%) in standard implants; 8 (11.94%) in immediate implants, 35 (23.03%) in early implants; 11 (20.37%) in implants with additional treatment, 32 (19.39%) in implants without additional treatment.

###  Associations between Implant Characteristics and Malpositioning

 According to the results of univariable logistic regression analysis, implants placed in the maxillary jaw exhibited a higher risk of malpositioning in comparison to those in the mandibular jaw (OR: 2.96; 95% CI: 1.43 to 6.14; *P* = 0.004). However, no significant correlation was observed between the timing of implant placement (immediate vs. early) (*P* *=*0.06), site of implant (anterior vs. posterior) (*P* *=*0.18), implant diameter (narrow vs. standard + wide) (*P* *=*0.80), implant length (extra-short + short vs. standard) (*P* *=*0.15), and additional treatment (yes vs. no) (*P* *=*0.88) with the occurrence of malpositioning (Table 2).

**Table 2:** Malpositioning occurrence and its association with implant characteristics

**Implant characteristics**	**Malpositioning**	**OR (95% CI)**	***P*** **-value**
Site of implant			
Anterior (n = 53)	7 (13.21%)	0.55 (0.23 to 1.32)	0.18
Posterior (n = 166)	36 (21.69%)	Reference	
Position of implant			
Maxilla (n = 113)	31 (27.43%)	2.96 (1.43 to 6.14)	0.004
Mandible (n = 106)	12 (11.32%)	Reference	
Implant diameter*			
Narrow (n = 80)	15 (18.75%)	0.91 (0.46 to 1.84)	0.80
Standard + wide (n = 139)	28 (20.14%)	Reference	
Implant length^#^			
Extra-short + short (n = 60)	8 (13.33%)	0.54 (0.24 to 1.25)	0.15
Standard (n = 159)	35 (22.01%)	Reference	
Timing of implant placement			
Immediate (n = 67)	8 (11.94%)	0.45 (0.20 to 1.04)	0.06
Early (n = 152)	35 (23.03%)	Reference	
Additional treatment			
Yes (n = 54)	11 (20.37%)	1.06 (0.49 to 2.29)	0.88
No (n = 165)	32 (19.39%)	Reference	

Data are expressed as no. (%), unless otherwise stated. Abbreviations: OR, Odds Ratio; CI, Confidence Interval Note: Malposition was defined as mesiodistal angulation ≥ 15° or inadequate proximity ( < 1.5 mm to adjacent teeth or < 3 mm to adjacent implants). *Implant diameter: extra-narrow ( < 3.0 mm), narrow ( ≥ 3.0 mm to < 3.75 mm), standard ( ≥ 3.75 to < 5 mm), and wide ( ≥ 5 mm) #Implant length: extra-short ( ≤ 6 mm), short ( > 6 mm to < 10 mm), standard ( ≥ 10 mm to < 13 mm), and long ( ≥ 13 mm)

 According to the results of multivariable logistic regression analysis, both the timing of implant placement (IIP vs. EIP) and the position of the implant (maxilla vs. mandible) were significant factors associated with malpositioning. Specifically, IIP demonstrated a lower risk of developing malpositioning in comparison to the EIP (adjusted OR: 0.38 (95% CI: 0.16 to 0.90; *P* *=*0.03; Nagelkerke R Square: 0.10; Cox & Snell R Square: 0.06).

## Discussion

 Ideal free-hand implant positioning is affected by several factors. The surgeon’s experience and the anatomical condition have been assessed as factors influencing the accuracy of a free-hand procedure.^14,15^ However, the impact of implant placement timing, specifically immediate versus early placement, has not been thoroughly documented as a contributing factor to implant malpositioning in prior studies. The present research examined two types of dental implant placement: the IIP group (30.6% of the sample) and the EIP group (69.4% of the sample). The current study did not investigate the association between sex and implant malposition. However, Safi et al.^13^ did not report a significant relationship between sex and implant malpositioning.^13^

 In the present study, in the immediate placement group, implants were most commonly inserted in the posterior maxilla, whereas in the early placement group, implants were most commonly inserted in the posterior mandible. Multivariable regression analysis demonstrated that the anteroposterior location of the implant did not significantly influence the likelihood of malpositioning. This observation concurs with a report by Safi et al.,^13^ who likewise found no statistically significant variation in malposition rates across different jaw sites.^13^

 Vasak et al.^16^ reported that while implants in the posterior maxilla had the lowest accuracy, those in the posterior mandible had the highest accuracy, followed by those in the posterior mandible.^16^ This was confirmed by other studies that also found that posterior regions of the jaws are less accurate, regarding implant positioning, than anterior sites.^17,18^ In the present study, implants in the maxilla were more prone to malposition than those in the mandible (OR: 2.96; 95% CI: 1.43 to 6.14; *P* = 0.004). This is consistent with previous studies that reported that specific quadrants and locations of the implant site significantly influence the direction and angular deviation of implant placement, as well as implant survival and success.^13,19,20^ However, Schnutenhaus et al.^14^ provided partial support for this relationship, as they found major deviations in the lower jaw.^14^

 In the present study, the implant diameters varied between the two groups. Regarding the prediction of implant malposition, implant diameter did not increase the incidence of malposition. This is consistent with the results of a study by Thanasrisuebwong et al.,^21^ which reported that implant diameters did not significantly affect placement deviations when a single posterior static surgical guide was used.^21^ Regarding the rate of implant malposition, the current study found no difference in the rate of implant malposition based on implant length. Additionally, this is consistent with earlier research that found no significant differences in malpositioning or implant size accuracy.^13,15^

 The timing of implantation showed that IIP cases were less likely to develop malposition than EIP cases. Compared to EIP, IIP showed a lower risk of malpositioning (adjusted OR: 0.38 (95% CI: 0.16 to 0.90; *P* *=*0.03). A possible explanation for this finding may lie in the alveolar socket’s role as a natural anatomical guide for accurate implant positioning, particularly with respect to angulation and orientation relative to adjacent structures. Another reason for this finding was that all IIPs were true indicators of this approach, with adequate apical bone available for primary stability and overall ideal anatomic conditions. Consistent with our results, Choi et al.^15^ examined the timing of implant placement and its impact on accuracy, finding that immediate implantation achieved higher accuracy and greater mesiodistal positioning accuracy.^15^ Schnutenhaus et al.^14^ emphasized the significance of implantation timing as an additional influencing factor, noting that its timing significantly affected the accuracy of free-hand implant placement. Their findings indicated that early implant placement (Type 3) exhibited higher deviations in both angulation and mesiodistal direction compared to late implant placements (Type 4).^14^ They reported that one explanation could be the degree of mineralization in the newly formed bone. This is consistent with the results of our study, which showed less accuracy in early implant placement.

 This study has several limitations that should be acknowledged. First, due to its cross-sectional design, case selection bias cannot be fully excluded, as the choice of immediate versus early placement was clinically driven rather than randomized; however, to address this limitation, regression analysis was used to account for related confounders. Moreover, because the implants were placed by multiple senior residents and the sample was obtained through consecutive (non-random) sampling, operator-related variability and selection bias cannot be fully excluded in our study. Additionally, this study used two-dimensional radiography data, which inherently limits the assessment of all aspects of malpositioning. Therefore, only the implant’s mesiodistal axial angulation relative to the bone crest and the distance between the implant and adjacent tooth or implant were measured as malpositioning criteria. We were unable to assess malpositioning in terms of proximity and angulation separately. Only the overall malpositioning rate was reported, as the sample size for evaluating proximity alone was insufficient. Future studies with larger samples are recommended to allow a more precise analysis of each component of malpositioning.

 Other malpositioning factors, such as faciolingual angulation, which might be more common specifically in IIP cases, and apicocoronal dimension, were not evaluated. Precise apicocoronal implant positioning is influenced by soft tissue height;^22^ however, as this parameter cannot be reliably assessed using periapical radiographs, it was excluded from measurement in this study.

 The rationale for employing parallel periapical (PA) radiography was twofold. Firstly, it aligns with the standard clinical workflow following implant placement. Secondly, it provides an effective initial screening method for identifying possible implant malposition. For a definitive diagnosis and precise measurement of malposition errors—particularly those involving invasion of critical anatomical structures—CBCT imaging is required. However, due to the significant radiation dose of CBCT, its use might be prudently limited to cases where initial PA radiography indicates a potential malposition or when supported by clinical evidence such as persistent pain or signs of infection. To the best of our knowledge, the influence of implant placement timing, particularly between immediate and early placement, has been inadequately documented as a factor contributing to implant malpositioning in previous studies. The present research demonstrated higher accuracy in implant positioning with immediate implant placement. For future investigations, prospective trials and studies incorporating digital planning and guided surgery are warranted to determine whether digital-assisted approaches can further improve IIP positioning accuracy.

## Conclusion

 Within the limitations of this cross-sectional study, multivariable regression indicated that both the timing and position of implant placement were predictors of malpositioning. IIP demonstrated a lower risk of malpositioning compared with EIP, while maxillary placement remained a significant risk factor.

## Competing Interests

 The authors declare that they have no financial and non-financial competing interests concerning the publication of their work during submission.

## Data Availability

 All data regarding the methodology of the manuscript have been shared.

## Ethical Approval

 The present study was approved by the Ethics Committee of Tehran University of Medical Sciences (ID: IR.TUMS.DENTISTRY.REC.1402.071).
